# Multimodal cell-cell communication driving CD8^+^ T cell dysfunction and immune evasion

**DOI:** 10.3389/fimmu.2025.1691746

**Published:** 2025-11-05

**Authors:** Liping Chen, Qianping Huang, Peipei Zhou

**Affiliations:** ^1^ The First Affiliated Hospital of Guangzhou Medical University, State Key Laboratory of Respiratory Diseases, Guangzhou, Guangdong, China; ^2^ Guangzhou National Laboratory, Bio-Island, Guangzhou, Guangdong, China

**Keywords:** tumor microenvironment, CD8^+^ T cell, multimodal cell-cell communication, suppression, dysfunction

## Abstract

Effective anti-tumor immunity critically depends on functional CD8^+^ T cells, yet in almost all solid tumors, these cells become dysfunctional, exhausted, or spatially excluded. This breakdown of immune surveillance arises not only from cell-intrinsic T cell exhaustion but also from multimodal communication among tumor, stromal, and immune cells within the tumor microenvironment (TME). This communication is mediated not only through direct receptor-ligand interactions but also through a suite of indirect mechanisms, such as metabolic competition, secretion of immunosuppressive metabolites and cytokines, extracellular vesicle exchange, and even mitochondrial transfer via tunneling nanotubes or membrane transfer through T cell trogocytosis. Together, these suppressive interactions impair CD8^+^ T cell metabolism, effector function, and persistence, thereby enabling tumor immune evasion. In this review, we summarize current understanding of how multimodal cell-cell communication, including immune checkpoints, metabolic reprogramming, and stromal crosstalk, cooperatively drive CD8^+^ T cell dysfunction. We also highlight emerging therapeutic strategies aimed at rewiring these suppressive networks, with emphasis on translational potential. A deeper understanding of the spatial, molecular, and metabolic context of CD8^+^ T cell suppression offers new avenues to enhance the efficacy of cancer immunotherapies.

## 1 Introduction

CD8^+^ cytotoxic T lymphocytes (CTLs) are central mediators of anti-tumor immunity, capable of directly eliminating malignant cells through perforin-granzyme release and Fas-FasL signaling (1, 2). Their activation requires tumor antigens presentation by dendritic cells (DCs), co-stimulation signals (e.g., CD28-B7), and pro-inflammation cytokines [e.g., interleukin (IL)-12, interferon-gamma (IFN-γ)], leading to clonal expansion and cytotoxic effector functions acquisition (3). Upon antigen-specific activation, CTLs proliferate and differentiate into two major subsets: effector CD8^+^ T cells, characterized by high expression of granzyme, perforin, and IFN-γ, which eliminate target tumor cells; and memory CD8^+^ T cells that possess self-renewal and multilineage differentiation capacities, providing a cellular reservoir for long-term immune surveillance (4, 5).

Under chronic antigen exposure, however, CTLs gradually lose effector function and upregulate inhibitory receptors such as programmed cell death protein 1 (PD-1) and cytotoxic T-lymphocyte associated protein 4 (CTLA-4), this dysfunctional state is termed T cell exhaustion (3). This trajectory of CD8^+^ T cell differentiation and dysfunction proceeds through successive stages: naïve T cells → activated T cells → stem-like progenitor of exhausted T cells (Tpex) → effector-like or intermediate exhausted T cells → terminal exhausted T cells (6, 7). TME provides spatial niches that critically shape this progression (8). Tertiary lymphoid structures (TLS) and perivascular regions, enriched with DCs, maintain TCF1^+^ Tpex cells, which preserve responsiveness to immune checkpoint blockade (ICB) (9–12). In contrast, tumor margins are enriched with CD103^+^ tissue-resident memory T cells (Trm) associated with favorable patient prognosis, while the immunosuppressive and hypoxic tumor core drives T cells towards terminal exhaustion, reinforced by persistent antigen exposure (13–15).

This dysfunctional state is further exacerbated by immunosuppressive factors in the TME, including tumor‐associated macrophages (TAMs, e.g., IRF8^+^) (16) and inhibitory cytokine networks (17), ultimately impairing antitumor immunity. Preclinical and clinical studies consistently demonstrate that in solid tumors, CD8^+^ T cells become functionally exhausted and metabolically impaired due to persistent antigen exposure and immunosuppressive mechanisms within the TME (18, 19). These mechanisms include direct inhibition by tumor and stromal cells, as well as indirect suppression via metabolic competition and soluble mediators, collectively impairing CD8^+^ T cell function and antitumor immunity (8, 20, 21).

A critical axis of immune evasion involves direct cell-to-cell interactions that drive CD8^+^ T cell dysfunction. Tumor cells exploit a repertoire of inhibitory ligands [e.g., PD-L1, B7 homolog 3 (B7-H3), and human leukocyte antigen (HLA)-E)] to engage checkpoint receptors [PD-1, Lymphocyte-activation gene 3 (LAG-3), Natural killer group 2 member A (NKG2A)] on T cells, thereby blunting TCR signaling and cytotoxicity activity. Immune cells such as regulatory T cells (Tregs) and myeloid-derived suppressor cells (MDSCs) further suppress CTLs through mechanisms including CTLA-4-mediated blockade of co-stimulation and PD-L1 expression. Cancer-associated fibroblasts (CAFs) reinforce this suppression both by expressing ligands such as PD-L1 and carcinoembryonic antigen-related cell adhesion molecule 1 (Ceacam-1), and by physically restricting CD8^+^ T cells infiltration into tumor niches. Together, these interactions highlight the complexity of contact-dependent immunosuppression and underscore the limitations of current checkpoint blockade therapies.

Beyond direct contact, the TME imposes indirect suppression through metabolic hijacking, stromal crosstalk, and biochemical perturbations. Tumor cells aggressively outcompete T cells for essential nutrients including glucose and arginine, while releasing immunosuppressive metabolites such as lactate, adenosine and kynurenine. Extracellular vesicles, tunneling nanotubes and T cell trogocytosis further exacerbate suppression by transferring inhibitory cargos, such as dysfunctional mitochondria, inhibitory miRNAs, or even membrane fragments, to T cells. Meanwhile, cytokines (e.g., TGF-β) and ions (e.g., Mg²^+^, ammonia) disrupt T cell metabolism, signaling and epigenetic programming. Stromal components such as CAFs and MDSCs amplify these effects by remodeling the extracellular matrix, secreting suppressive cytokines, and inducing hypoxia. Collectively, these processes create a hostile metabolic and structural niche that sustains T cell dysfunction.

**Figure 1:**
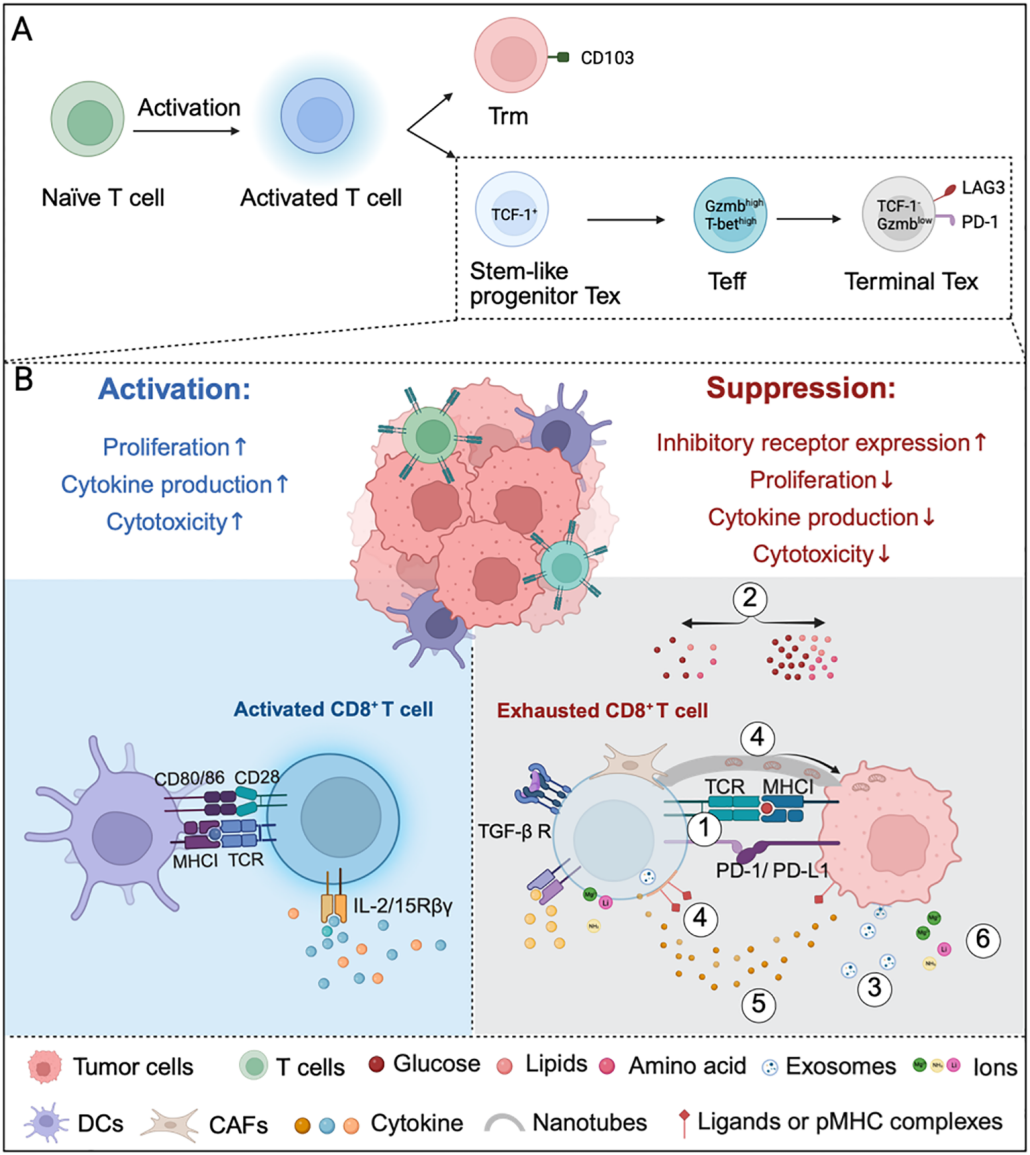
Multifaceted regulation of CD8^+^ T cell function within the TME. **(A)** Differentiation and exhaustion of CD8^+^ T cells under chronic antigen stimulation. Effector-like or intermediate exhausted T cells (Teff); Exhausted T cells (Tex); Tissue-resident memory T cells (Trm). **(B)** The TME exerts dual effects on CD8^+^ T cells: it can promote T cell activation and effector functions, while simultaneously driving exhaustion and dysfunction. Left panel (Activation): Dendritic cells prime CD8^+^ T cells through integrated signals, which collectively enhance T cell proliferation, migration, differentiation, cytokine production, and cytotoxic capacity. Right panel (Suppression): Tumor cells suppress CD8^+^ T cell function through multiple mechanisms: (1) immunosuppressive ligand-receptor interactions [programmed cell death ligand 1 (PD-L1)-PD-1, transforming growth factor β (TGF-β)-TGF β receptor (TGF-βR)]; (2) nutrient competition (glucose, lipids, and amino acids); (3) tumor-derived exosomes; (4) intercellular material transfer via nanotubes and trogocytosis; (5) cytokines; and (6) release of immunosuppressive cytokines or metabolites (Mg2^+^, lithium, and ammonia). These inhibitor cues collectively drive upregulation of checkpoint receptors, diminished proliferation, and self-renewal capacity, reduced cytokine production, and impaired cytotoxicity, ultimately driving CD8^+^ T cells toward exhaustion. Image created with bioRender.com, with permission. Created in BioRender. Zhou, P. (2025) https://BioRender.com/e66x2mi.

These multimodal pathways act synergistically to impair CD8^+^ T cell cytotoxicity and persistence, and spatial access into tumors, ultimately enabling immune evasion. Overcoming this coordinated suppression remains a major challenge in current cancer immunotherapy. In this review, we summarize recent advances in understanding the mechanisms of multimodal cell-cell communication, including immune checkpoint signaling, metabolic interference, and stromal crosstalk, that collectively drives CD8^+^ T cell dysfunction (**Figure 1**). We further discuss emerging therapeutic strategies designed to disrupt these suppressive networks and restore anti-tumor immunity, with particular attention to combinatorial approaches with translational potential. A precise understanding of the spatial and molecular dynamics of CD8^+^ T cell suppression will be pivotal for overcoming resistance to current immunotherapies.

## 2 Direct cell-to-cell interactions suppressing CD8^+^ T cell function

**Figure 2:**
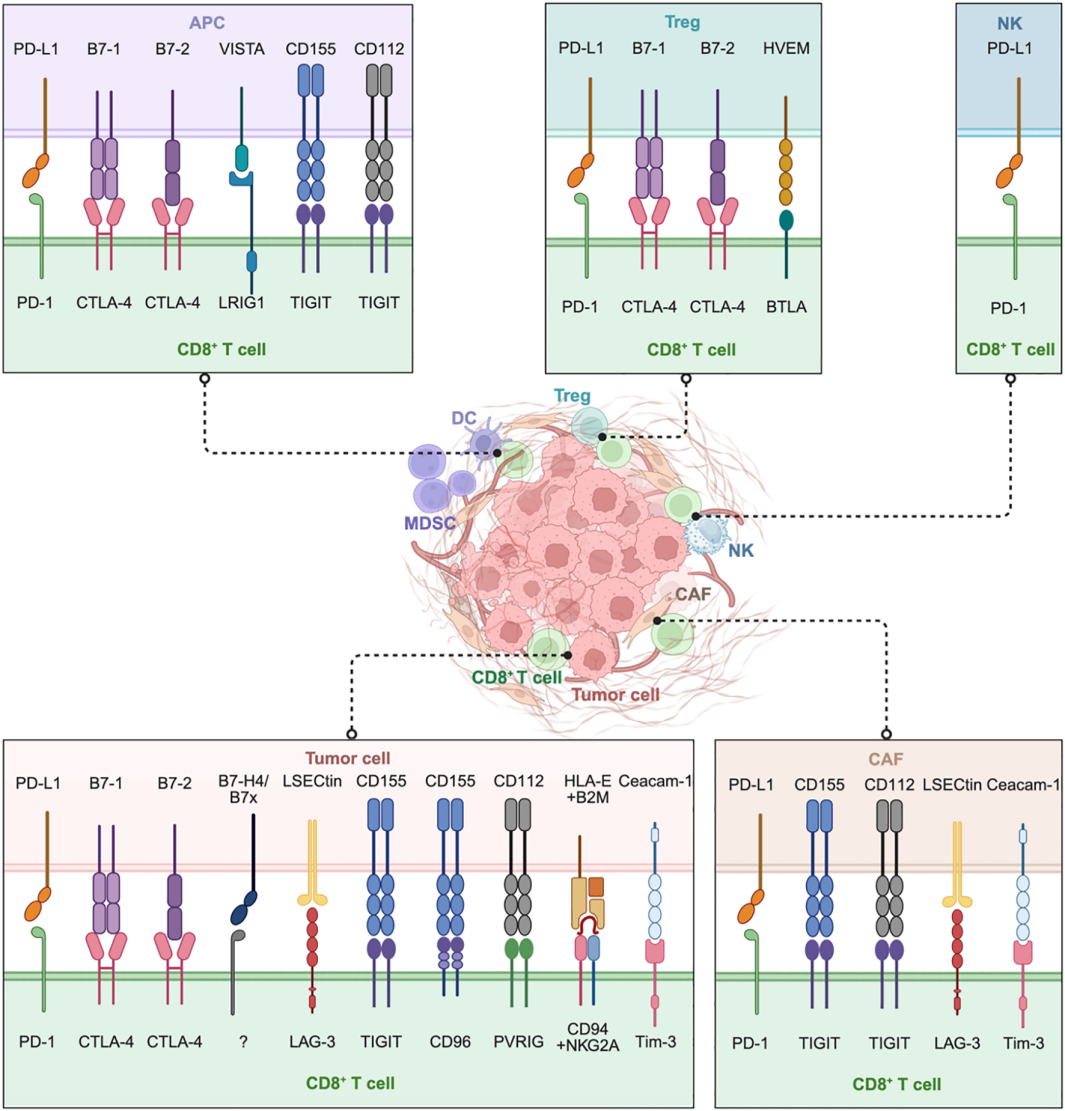
Direct cell-to-cell contact plays a critical role in the suppression of CD8^+^ T cells within the TME. Tumor cells inhibit CTLs by engaging inhibitory ligands with corresponding receptors, while multiple immune cells, including antigen-presenting cells (APCs), regulatory T cells (Tregs), NK cells (NKs), and specialized CD8^+^ Tregs, further suppress CD8^+^ T cells through checkpoint molecules like PD-1, CTLA-4, and VISTA. Cancer-associated fibroblasts (CAFs) uniquely contribute by engaging in direct inhibitory signaling (such as PD-L1-PD-1, Ceacam-1-Tim-3) and by imposing physical barriers that restrict T cell infiltration. Collectively, this intricate intercellular communication network drives CD8^+^ T cell dysfunction and exhaustion. Targeting these specific interactions, particularly beyond PD-1 and CTLA-4 (e.g., LAG-3, TIGIT, VISTA, PVRIG, CD96, NKG2A) and disrupting CAF-mediated suppression represent promising approaches to great reinvigorate CD8^+^ T cell anti-tumor responses. Image created with bioRender.com, with permission. Created in BioRender. Zhou, P. (2025) https://BioRender.com/kjhqxg8.

The direct interaction between CD8^+^ T cells and other cells in TME, including tumor cells, other immune cells and CAFs, is crucial for shaping anti-tumor immune responses. Direct contact through receptor ligand engagement and immunological synapses regulates CD8^+^ T cell activation, effector function, and exhaustion. While stimulatory signals enhance cytotoxicity, some interaction induced inhibitory pathways blunt TCR signaling, cytokine production, and proliferation. This section reviews how tumor cells, immune cells, and CAFs suppress CD8^+^ T cells function through surface expressed inhibitory molecules and checkpoint receptor-ligand interactions (**Figure 2**).

### 2.1 Tumor cell-to-CD8^+^ T cell interactions

Tumor cells directly inhibit infiltrating CD8^+^ T cells by engaging multiple inhibitory ligands. The PD-1-PD-L1 axis remains a dominant pathway: IFN-γ produced by activated T cells induces PD-L1 expression on tumor cells (22), which in turn binds PD-1 on CD8^+^ T cells, delivering potent inhibitory signals that attenuate TCR signaling (e.g., reduced ZAP70 phosphorylation), cytokine secretion (e.g., IFN-γ), and cytotoxic activity, ultimately driving CD8^+^ T cells into a dysfunctional state. Similarly, B7 ligands B7-1 (CD80) and B7-2 (CD86) on tumor cells engage CTLA-4 on activated CD8^+^ T cells, outcompeting CD28 and thereby blocking co-stimulatory signals required for T cell activation, leading to CD8^+^ T cell anergy.

Beyond these classical checkpoints, emerging ligand-receptor pathways are increasingly recognized. B7x-B7-H4, a member of the B7 family broadly expressed across tumors, which binds unidentified inhibitory receptor on activated, but not resting CD8^+^ T cells (23–25). B7-H4 inhibits CD8^+^ T cell responses at an early stage primarily by arresting cell cycle progression, suppressing TCR signaling, and reducing IL-2 production (26). Liver and lymph node sinusoidal endothelial cell C-type lectin (LSECtin), expressed in the liver and on multiple tumor types (e.g., melanoma), suppresses anti-tumor immunity by binding LAG-3 on CD8^+^ T cells, where its KIEELE motif has been identified as structurally and functionally essential for LAG-3’s inhibitory capacity. LAG-3 signaling inhibits effector T cell function by associating with CD3, where co-engagement suppresses proliferation, IFN-γ secretion, and calcium mobilization (27, 28).

T cell immunoglobulin and ITIM domain (TIGIT), an Ig superfamily member specifically expressed in immune cells, binds CD155 on tumor cells, directly inhibiting effector CD8^+^ T cell function (27). CD96, which also binds CD155, antagonizes the activating receptor CD226. Although CD96-mediated intracellular signaling remains incompletely characterized, its cytoplasmic ITIM domain suggests inhibitory potential (29). Notably, CD155^hi^ lung adenocarcinoma (LUAD) cells dramatically reduce IFN-γ production in CD8^+^ T cells, thereby suppressing antitumor immunity (30). Poliovirus receptor-related protein 2 (PVRL2), also known as CD112, expressed by tumor cells and tumor-associated myeloid cells, binds the late-induced inhibitory receptor PVRIG (CD112R) on activated CD8^+^ T cells. The PVRL2-PVRIG axis, mediated by PVRIG’s ITIM domain, diminishes IL-12 receptor expression, suppresses cytotoxicity, and promotes CD8^+^ T cell exhaustion (31, 32).

Additional interactions further reinforce this suppressive network. HLA-E-Qa-1^b^ complexes, presenting specific peptides processed by endoplasmic reticulum aminopeptidase 1-2 (ERAP1-2), engage the inhibitory natural killer cell group 2 member A (NKG2A)-CD94 heterodimer on a subset of CD8^+^ tumor-infiltrating lymphocytes (TILs), leading to suppression of TCR signaling and consequent impairment of cytotoxic effector function (33, 34). Ceacam-1-Tim-3 interactions have also been implicated, although current support comes primarily from clinical evidence rather than experimental validation (35).

Collectively, these inhibitory dyads converge to restrain CD8^+^ T cell cytotoxicity and persistence, underscoring the importance of multi-targeted checkpoint blockade.

### 2.2 Immune cell-to-CD8^+^ T cell interactions

Multiple immune cell populations within the TME suppress CD8^+^ T cell function through direct contact. Antigen-presenting cells (APCs), including DCs and macrophages, inhibit CD8^+^ T cells through classic immune evasion pathways, like PD-L1-PD-1 axis (36–39). APCs also express VISTA (V-domain immunoglobulin suppressor of T cell activation), functioning as a ligand for immunoglobulin-like domains 1 (LRIG1) on CD8^+^ T cells in a “trans” configuration, contributing to T cell inhibition and quiescence (40–42). Furthermore, constitutive expression of CD80-CD86 on APCs allows binding of CTLA-4 on activated CD8^+^ T cells (43, 44). CTLA-4 not only transmits intrinsic inhibitory signals but also, on Tregs, mediates the trans-endocytosis and degradation of CD80-CD86 from the APC surface, thereby limiting co-stimulation for other T cells (43, 45). Follicular dendritic cells (FDCs) express CD112 and CD155, which engage TIGIT on TILs, promoting a dysfunctional state characterized by high co-expression of PD-1, and diminished production of IFN-γ, tumor necrosis factor-alpha (TNF-α), and IL-2 (46). Natural Killer (NK) cells upregulate PD-L1 upon tumor recognition and IL-18 stimulation, generating PD-L1^hi^ NK cells that directly suppress CD8^+^ T cell proliferation in PD-L1-PD-1-dependent manner (47). CD45RA^−^ CCR7^−^ (C-C motif chemokine receptor 7) Tregs exhibit upregulated CD80/CD86 expression alongside reduced HLA-DR, enabling potent suppression of CD8^+^ T cell function through dual mechanisms: IL-10 secretion and cell-contact-dependent inhibition mediated by CD80/CD86-CTLA-4 interaction, as evidenced by diminished IFN-γ, granzyme B production, and proliferation (48). Herpes virus entry mediator (HVEM, also TNFRSF14), a member of the TNF receptor superfamily expressed by both immune and non-immune cells that is frequently upregulated in malignancies, engages B and T lymphocyte attenuator (BTLA) on T cells to trigger co-inhibitory signaling, thereby suppressing TCR-mediated activation and impairing cytotoxic effector function (49, 50). Intriguingly, CD8^+^ T cells themselves may acquire suppression function. For example, a subset of CD8^+^ T cells, identified in humans as CD8^+^HLA-DR^+^ T cells, can adopt regulatory functions, further constraining effector responses (51). LRIG1, expressed on CD8^+^ T cells, interact with VISTA in cis or trans to suppresses anti-tumor immunity by inducing quiescence in CD8^+^ T cells and limiting the development of effector T cells from progenitor and memory-like cells (40). In summary, the effectiveness of CD8^+^ T cells in controlling tumors are significantly limited by an inhibitory interaction established immunosuppressive network in the TME.

### 2.3 CAFs-to-CD8^+^ T cell interactions

CAFs suppress CD8^+^ T cell function through both checkpoint signaling and structural modulation of the TME. CAFs frequently express PD-L1 (52), reciprocally upregulated through crosstalk with tumor cells via contact or soluble factors, which directly binds PD-1 on CD8^+^ T cells and correlates with poor prognosis in cancers like esophageal carcinoma. Like tumor cells and FDCs, CAFs also express CD155 and CD112, engaging TIGIT on TILs. TIGIT^+^ PD-1^+^ T cells exhibit reduced IFN-γ, TNF-α, and IL-2 production and impaired cytotoxicity, marking dysfunctional CD8^+^ T effector memory cells (T_EM_) cells. Dual blockade of TIGIT and PD-1 reverses this exhaustion, restoring antitumor responses (46). In hepatic tissues, LSECtin on hepatic CAFs engages LAG-3 on CD8^+^ T cells via the KIEELE motif, recruiting inhibitory signals through CD3 to suppress proliferation, IFN-γ, and calcium flux, dampening antitumor immunity (27). Moreover, CAFs express other immunosuppressive ligands: Ceacam-1 binds TIM-3 on CD8^+^ T cells, reinforcing exhaustion (27, 53). Beyond checkpoint ligands, CAFs remodel the extracellular matrix, restrict CD8^+^ T cells infiltration, and secret cytokines and exosomes that further impair function.

Through these diverse roles, CAFs act as key regulators of immune exclusion and resistance to immunotherapy. Targeting CAFs-CD8^+^ T cells interactions represents a promising strategy for successful cancer immunotherapies combination with checkpoint blockade.

### 2.4 Therapeutic strategies targeting direct cell-cell interactions

Immune checkpoints such as PD-1 and CTLA-4 are critical regulators of immune tolerance, preventing excessive immune activation. Tumors exploit this mechanism through ligand overexpression (e.g., PD-L1) to suppress T-cell function and facilitate immune escape. ICB therapies targeting PD-1-PD-L1, CTLA-4, and LAG-3 have significantly improved survival in multiple cancers (54, 55). However, complete response rates remain limited (56), largely due to tumor heterogeneity and the complexity of the immunosuppressive in TME, underscoring the need for stratified and context-specific immunotherapy approaches (15).

The functional state of CD8^+^ T cells, which serve as the core effector cells in antitumor immunity, is not shaped by a single signal but instead by integrated crosstalk with diverse cell populations in the TME (57, 58). Accordingly, immunotherapy strategies are shifting from a T cell-centric focus toward approaches that modulate the cellular interactions within the TME to promote effective antitumor immunity. The central therapeutic goal is to enhance T cell recognition and effector function while simultaneously blocking tumor immune evasion pathways.

Immune checkpoint inhibitors (ICIs) represent the most direct strategy (49). Anti–PD-1-PD-L1 antibodies restore effector functions of CD8^+^ T cells (such as cytokine secretion and cytotoxicity) by disrupting PD-L1-PD-1 inhibitory axis (49). Beyond classical ICIs, novel checkpoints such as TIGIT have been identified (59, 60). While anti-TIGIT monotherapy or combination therapy with anti–PD-1 has shown potential in some clinical trials, these approached remain insufficient to fully reinvigorate CD8^+^ T cells, particularly in patients with advanced or high tumor burden (61, 62). To enhance TIGIT-targeted immunotherapy, combination regimens are being developed, including anti–CTLA-4 or anti-vascular endothelial growth factor (VEGF) agents in triple blockade (e.g., TIGIT + PD-1-PD-L1 + CTLA-4 or + VEGF), or combinations with chemotherapy (59). In addition, multiple bispecific and trispecific antibodies have also entered clinical development, showing preliminary potential in overcoming resistance.

Beyond checkpoint inhibition, targeting interactions between CD8^+^ T cells and other immune cells offers additional therapeutic avenues (57). For example, anti-CTLA-4 antibodies (e.g., ipilimumab) function in part by depleting intertumoral Tregs, thereby relieving suppression on CD8^+^ T cells (63). Additionally, combination therapy with doxorubicin and IL-12 has been shown to shift receptor signaling in tumor infiltrating CD8^+^ T cells toward immunostimulatory pathways while reducing Treg infiltration, thus enhancing local effector activity (64).

CAFs present another major challenge to restrict CD8^+^ T cell infiltration and function by constructing both physical and biochemical barriers (65). Overcoming CAF-mediated immunosuppression is thus critical for restoring CD8^+^ T cell-mediated antitumor activity (66). In triple-negative breast cancer (TNBC), CAFs are particularly important therapeutic targets. Huo et al. engineered a CAF-targeted nanosystem co-loaded with a TGF-β inhibitor (LY3200882) and PD-L1 siRNA. Upon matrix metalloproteinase-2 (MMP2)-responsive release, LY3200882 preferentially modulates CAF activity, reducing extracellular matrix deposition and enhancing T-cell infiltration. Simultaneously, PD-L1 siRNA downregulates PD-L1 expression in both tumor cells and CAFs. This dual-action strategy effectively reverses CAF-driven immunosuppression, remodels the TME, and suppresses TNBC progression (67).

## 3 Indirect suppression via TME

The TME exerts profound indirect suppression on CD8^+^ T cell responses, orchestrating a complex network of metabolic, biochemical and structural barriers that shape anti-tumor immunity. Mounting evidence indicates that tumors co-opt multifaceted pathways, including metabolic reprogramming, cytokine induction, receptor modulation, and immune checkpoint activation, to systemically impair CD8^+^ T cell effector function, thereby fostering tumor progression. These immunosuppressive circuits are increasingly recognized as critical drivers of tumor immune evasion, positioning them as attractive therapeutic targets for restoring anti-tumor immunity. This section focuses on indirect TME-driven suppression, delineating how tumor cells and stromal elements orchestrate CD8^+^ T cell suppression through metabolic competition (e.g., nutrient deprivation), intercellular communication (eg. exosomes, tunneling nanotubes, or trogocytosis), and microenvironmental perturbations (eg. cytokine networks, ionic imbalances, or ammonia accumulation) (**Table 1**). Collectively, these mechanisms establish an immunosuppressive niche that subverts CD8^+^ T cell surveillance and therapeutic efficacy.

**Table 1:** Indirect regulation of CD8^+^ T cell dysfunction and exhaustion by the TME.

Classification of indirect suppression		Mechanisms and conclusions
Tumor-CD8^+^ T cell nutrient competition	Glucose	In the tumor microenvironment, cancer cells or myeloid cells (68) outcompete CD8^+^ T cells for glucose via the Warburg effect (69), leading to lactate accumulation (70, 71), acidosis, and metabolic stress (72), by upregulate glucose transporters GLUT1 and GLUT3 (73, 74), or elevated glucose metabolism (75). This nutrient deprivation impairs T cell mitochondrial function, mTOR signaling (76, 77), and effector responses, while promoting exhaustion markers (PD-1, LAG-3) and epigenetic dysfunction (72, 78–80). Targeting this metabolic competition may enhance immunotherapy efficacy (81).
	Lipids	Very-long-chain acyl-CoA dehydrogenase (VLCAD) (82), long-chain fatty acids (LCFAs) (83), arachidonic acid (82), lipid droplet (84), prostaglandin E2 (85), or PCSK9–63 impair CD8^+^ T cell activity.
	Amino Acid	Depletion of arginine (86–88), alanine (89), glutamine (89), tryptophan (90), or accumulation of adenosine (91, 92), L-ornithine (90) suppress T cell activation, proliferation, and cytokine production.
Exosomes		Exosomes inhibit the function of CD8^+^ T cells and enhance their apoptosis by delivering immunosuppressive molecules (e.g., cytokines (93, 94), regulatory miRNAs (93, 95, 96), and metabolic modulators (97)) or transmitting signals via direct contact (93, 94).
Nanotubes and Trogocytosis		Transfer of mitochondria (98), nutrients depletion (99) or “self-inhibition” (100) through acquisition of inhibitory ligands or “antigen loss” (101, 102), collectively rewire T cell metabolism and blunt antigen recognition, thereby hindering CD8^+^ T cell function.
Cytokines		Inhibitory cytokines predominantly impair CD8^+^ T cell proliferation and effector function, including TGF-β (103), IL-2 (104), IL-6 (105), IL-18R (106), IL-27 (107), or IL-10 (108, 109) and IL-35 (110).
Ions and Metabolites		Dysregulated Mg^2+^ (111), Lithium (112) and ammonia (113) levels interfere with T cell function and mechanisms.

### 3.1 Tumor-CD8^+^ T cell nutrient competition

The availability of nutrients within the TME has emerged as a pivotal determinant of CD8^+^ T cell function. Compelling evidence indicates that enhanced nutrient uptake, glycolytic flux, and oxidative metabolism collectively potentiate CD8^+^ T cell proliferation and effector differentiation within tumors. This metabolic adaptation is essential for sustaining anti-tumor responses. Nevertheless, the TME frequently imposes profound metabolic constraints, including nutrient deprivation and lipid accumulation, that directly impair CD8^+^ T cell effector responses and immune surveillance. Strategies to overcome these barriers show therapeutic promise.

#### 3.1.1 Glucose

**Figure 3:**
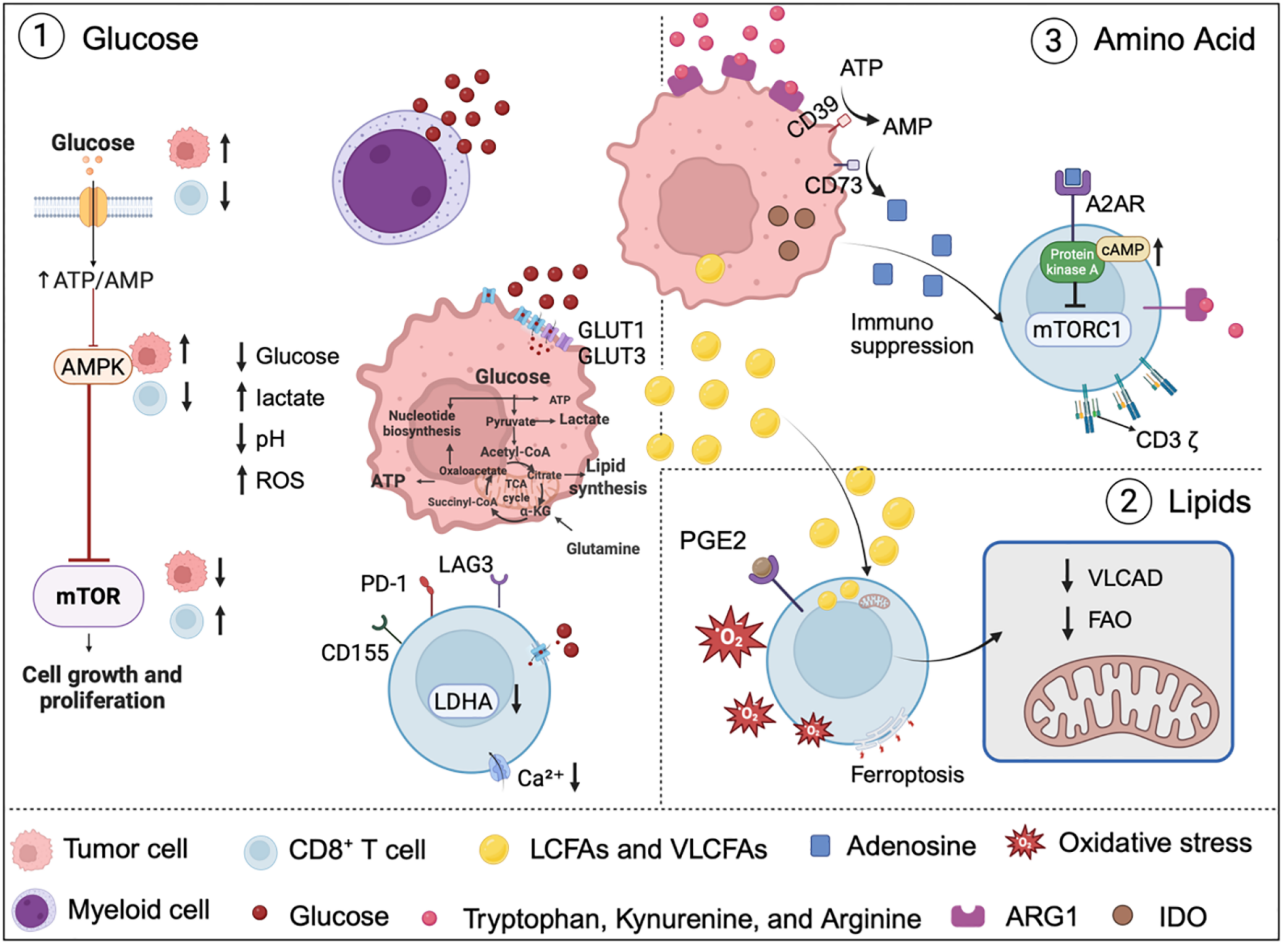
Metabolic Reprogramming in the TME Driving CD8^+^ T Cell Dysfunction. Glucose, lipid, and amino acid metabolism in the TME collectively impair CD8^+^ T cell function through nutrient competition, metabolite accumulation, and inhibitory signaling (1). Tumor cells and myeloid cells mediated glucose uptake and lactate accumulation suppress glycoses and mTOR activity in T cells. (2) Accumulation of long-chain fatty acids (LCFAs) and lipid abnormalities within T cells causes lipotoxicity and mitochondrial dysfunction. (3) Amino acid depletion by enzymes such as arginase 1 (ARG1) and IDO disrupts TCR signaling and generates immunosuppressive metabolites such as adenosine. These metabolic pathways collectively drive T cell dysfunction and represent potential therapeutic targets. Image created with bioRender.com, with permission. Created in BioRender. Zhou, P. (2025) https://BioRender.com/8h0fjul.

Glucose metabolism plays a pivotal role in the TME, impacting both tumor progression and the functional capabilities of TILs (**Figure 3**). Tumor cells exploit the Warburg effect, consuming glucose and releasing lactate, which drives extracellular acidosis, hypoxia, disordered vasculature, and dense extracellular matrix within the TME (69, 73, 79, 114, 115). This nutrient competition restricts glucose availability to TILs, resulting in mitochondrial dysfunction and altered lipid metabolism, ultimately hindering T cell effector function and persistence. To sustain growth, tumor cells upregulate glucose transporters such as GLUT1 and GLUT3, and avidly consuming glucose and glutamine to promote T cell exhaustion and immune evasion (73, 74). In renal cell carcinoma, elevated tumor glycolysis corelates with reduced effector CD8^+^ T cells (75). Nutrient deprivation triggers AMP-activated protein kinase (AMPK) activation, while suppressing mTOR thereby disrupting T cell differentiation (116). Moreover, dysregulation of glucose metabolism through pathways such as PI3K/AKT/mTOR signaling further impacts T cell activation, Ca²^+^ signaling, and O-GlcNAcylation, all of which are essential for T cell effector function (76, 77, 117).

Emerging evidence challenges the notion that immune dysfunction arises solely from tumor-driven nutrient deprivation. Reinfeld et al. demonstrated that myeloid cells, rather than T cells or tumor cells, exhibit the highest glucose uptake, while tumor cells preferentially rely on glutamine metabolism (68). These distinct metabolic programs are governed by intrinsic cellular programming mechanisms including differential mTORC1 activity and metabolic gene expression, rather than extracellular nutrient competition (68). Moreover, inhibiting glutamine metabolism was further shown to enhance glucose uptake across multiple cell types, suggesting a feedback mechanism between glucose and glutamine utilization. These findings emphasizes that immune metabolic dysfunction in the TME is shaped not only by nutrient deprivation but also by cell type-specific cellular metabolic programming, providing novel directions for metabolism-based therapeutic strategies.

Beyond nutrient depletion, additional metabolic barriers, including lactate accumulation, acidic pH, hypoxia, and elevated ROS, further contribute to T cell dysfunction by reprogramming metabolism and upregulating immune checkpoint expression (72). Notably, PD-L1 blockade has been shown to enhance T cell infiltration and metabolic fitness in glycolysis-low tumors (78). Conversely, inhibition of lactate dehydrogenase (LDHA) impairs CD8^+^ T cell migration, proliferation, and effector functions (70), while blockade of OGR1 in melanoma restores CD8^+^ T cell cytotoxic activity (71).

Together, glucose dysregulation in the TME not only hinders T cell effector functions but also increases the immune checkpoint expression and exhaustion, constituting a key mechanism of tumor immune evasion. These insights underscore the therapeutic potential of reprograming glucose metabolism by enhancing T cell glycolytic capacity, restraining tumor glycolysis, or targeting glutamine-glucose metabolic crosstalk, to overcome metabolic barriers and enhance immunotherapeutic efficacy (81).

#### 3.1.2 Lipids

The interplay between lipids and CD8^+^ T cell dysfunction within the TME has attracted growing interest, revealing complex mechanisms by which lipid accumulation and metabolism shape anti-tumor immunity. Lipid metabolism dichotomizes into opposing immunomodulatory pathways within the TME: one suppresses CD8^+^ T cell effector function (118–120), while the other sustains or enhances CD8^+^ T cell activation (121). This section highlights the specific immunosuppressive lipids present in the TME and delineate the mechanisms by which they impair CD8^+^ T cell activity (**Figure 3**). For example, intrapancreatic CD8^+^ T cells exhibit downregulation of very-long-chain acyl-CoA dehydrogenase (VLCAD), exacerbating the accumulation of lipotoxic long-chain fatty acids (LCFAs) and VLCFAs (82). Metabolic reprogramming through enforced VLCAD expression enhanced intratumorally T cell survival and persistence in a pancreatic ductal adenocarcinoma (PDA) mouse model, overcoming a major immunotherapy hurdle (82). LCFAs such as palmitate impede CD8^+^ T cell proliferation and effector cytokine production (83). Among unsaturated fatty acids, oleic acid and linoleic acid exert divergent effects on tumor progression: linoleic acid reprograms tumor-infiltrating CD8^+^ T cells from an exhausted phenotype towards a memory-like state, potentiating their effector function (122). Arachidonic acid induces ferroptosis in tumor cells but may concurrently trigger ferroptosis in tumor-infiltrating CD8^+^ T cells (82). The TME induces lipid droplet accumulation in dysfunctional CD8^+^ TILs through acetyl-CoA carboxylase-mediated metabolic reprogramming (84). Prostaglandin E2 impairs IL-2 sensing in human CD8^+^ T cells, promoting oxidative stress and ferroptosis (85). Cholesterol and its derivatives critically modulate CD8^+^ T cell function in context-dependent manner: cholesterol enhances TCR signaling, yet tumor cells derived PCSK9 dysregulates CD8^+^ T cell cholesterol metabolism, thereby suppressing TCR signaling (123), while the oxysterol 27-hydroxycholesterol facilitates metastasis, an effect potently suppressed by CYP27A1 inhibition (124). Notably, in pancreatic tumors, CD8^+^ T cell accumulation of LCFAs impairs mitochondrial function and fatty acid catabolism, recapitulating the proliferative and cytokine defects observed upon *in vitro* palmitate treatment (82). Rather than serving as an energy source, these accumulated lipids impair mitochondrial function and induce transcriptional reprogramming of lipid metabolism pathways, ultimately hampering CD8^+^ T cell metabolic fitness and anti-tumor activity (82).

#### 3.1.3 Amino acid

The TME orchestrates a complex metabolic interplay where amino acid availability profoundly impacts the functionality of CD8^+^ T cells through diverse mechanisms (**Figure 3**). Amino acids serve as critical substrates for various cellular processes such as protein synthesis, epigenetic modifications (e.g., SAM-dependent methylation), and energy metabolism, making them highly contested resources between tumor cells and T cells. For example, in activated T cells, extracellular alanine is preferentially utilized for protein synthesis rather than catabolism. Arginine catabolism by arginase 1 (ARG1) and inducible nitric oxide synthase (iNOS) impairs TCR function by downregulating the CD3ξ chain expression (86). Moreover, ARG1-containing extracellular vesicles can traffic to draining lymph nodes, where their uptake by dendritic cells suppresses antigen-specific T-cell proliferation, as demonstrated in ovarian carcinoma models (87, 88). Adenosine further compromises T cell function and metabolic fitness through the A2AR/PKA/mTORC1 pathway, dampening both peripheral and tumor-infiltrating CD8^+^ T cells (91, 92). Alanine deprivation delays the activation of naive and memory T cells (125), although it has limited effects on T cell effector function. In contrast, glutamine deprivation restricts metabolic flexibility, while SLC7A11, a multi-pass transmembrane protein, driven cysteine depletion promotes oxidative stress (89). L-ornithine has been shown to suppress T cell functionality, as observed in murine models of chronic viral infection where altered expression of hepatic urea cycle enzymes results in L-ornithine accumulation, leading to the inhibition of virus-specific CD8^+^ T cell responses (126). Similarly, tryptophan depletion triggers GCN2-mediated stress responses that suppress mTOR signaling, further restricting T cell activity (90).

Collectively, these metabolic perturbations disrupt T cell activation, proliferation, and the production of effector molecules, thereby contributing to immunotherapy resistance. Targeting this metabolic axis offers novel therapeutic strategies, such as inhibiting ARG1 or GLS in combination with immune checkpoint blockade, may restore amino acid homeostasis and reinvigorate antitumor immunity. Such strategies highlight a promising frontier that integrates metabolic and immunological intervention to overcome treatment resistance.

### 3.2 Exosomes

In various cancers, exosomes derived from tumor cells or stromal cells carry molecular cargo that induces dysfunction or exhaustion of CD8^+^ T cells, thereby facilitating tumor progression and resistance to immunotherapy. Exosomes suppress CD8^+^ T cell function and promote their apoptosis through two primary mechanisms: (1) delivery of immunosuppressive molecules and (2) ligand-receptor interactions that trigger contact-dependent signaling.

In the first route, exosomes transport inhibitory factors including cytokines [e.g., TGF-β (93), IL-8 (94)], regulatory miRNAs [e.g., microRNAs (93, 95) and circRNA (96)], and metabolic modulators (97) (e.g., lactate dehydrogenase LDHA and lactate). These cargos collectively impair T cell activation, disrupt inflammatory signaling pathways (eg. STAT1-IFN-γ) and compromise glycolytic metabolism. In the second route, exosome surface ligands, including PD-L1 (127) and FasL, engage corresponding receptors on CD8^+^ T cells, driving exhaustion or apoptosis. Together, these coordinated immunosuppressive actions establish exosomes as critical mediators of T cell dysfunction in cancer, while also presenting potential therapeutic targets for enhancing immunotherapies. Recent studies demonstrate the breadth of this regulation. For example, Fan Xu et al. showed that IL-8 in exosomes derived from prostate cancer cells hyperactivates peroxisome proliferators-activated receptors (PPARα) in recipient CD8^+^ T cells, which downregulates GLUT1 and hexokinase 2 to reduce glucose utilization while upregulating Carnitine O-palmitoyltransferase 1 and peroxisomal acyl-coenzyme A oxidase 1 to enhance fatty acid catabolism, ultimately exacerbating CD8^+^ T cell starvation and promoting cellular exhaustion (94). Non-small cell lung cancer (NSCLC) cells release circUSP7 via exosome secretion, which upregulates SHP2 expression by sponging miR-934, thereby inhibiting CD8^+^ T cell secretion of IFN-γ, TNF-α, granzyme B, and perforin and ultimately suppressing CD8^+^ T cell function (128). Another example is the exosome circCCAR1, which is taken up by CD8^+^ T cells and induces CD8^+^ T cell dysfunction by stabilizing PD-1 protein (96). Collectively, these studies delineate a complex network whereby tumor and stromal cell-derived exosomes carry diverse molecular cargos, including circRNAs, cytokines and proteins, that induce CD8^+^ T cell dysfunction, in addition offering novel opportunities for therapeutic targets.

### 3.3 Nanotubes and trogocytosis

The contribution of nanotubes and trogocytosis in regulating CD8^+^ T cell function within the TME has become an emerging area, particularly regarding intercellular mitochondrial transfer and its consequences on T cell efficacy. Mitochondrial dysfunction in CD8^+^ T cells represents a fundamental driver of T cell exhaustion in tumor contexts, making these intercellular communication mechanisms highly relevant to tumor immune evasion.

Current evidence reveals that nanotube-mediated mitochondrial transfer exhibits dual functionality. On one hand, nanotubes can restore T cell metabolic activity by delivering functional mitochondria; on the other hand, tumor cells often exploit this progress to transfer dysfunctional mitochondria containing mutations or oxidative damage, thereby promoting T cell failure. The principal inhibitory mechanisms of nanotubes toward CD8^+^ T cells encompass metabolic subversion through mitochondrial hijacking (98) and nutrient deprivation (99). Using multimodal imaging and metabolic profiling, Tanmoy Saha et al. demonstrated that cancer cells hijack mitochondria from immune cells via tunneling nanotubes, simultaneously depleting immune cell function while metabolically empowering tumor cells (129). In contrast, Jeremy G. Baldwin et al. showed that bone marrow stromal cells transfer healthy mitochondria to CD8^+^ T cells through intercellular nanotubes, thereby restoring CD8^+^ T cell function and promoting anti-tumor responses (98). Together, these findings highlight the complex, context-dependent role of nanotubes in immune regulation and underscore their potential as therapeutic targets in cancer immunotherapy.

Trogocytosis, the direct transfer of membrane fragments and regulatory molecules during cell-cell contact, also play a crucial role on T cell function. In the TME, CD8^+^ T cells that acquire inhibitory molecules from APCs or tumor cells can undergo suppression of cytokine production and proliferation through reverse signaling (45). Mechanistically, trogocytosis in CD8^+^ T cells, where they acquire inhibitory ligands or pMHC complexes, can promote immune evasion, leading to T cell exhaustion mainly through “self-inhibition” (100) and “antigen loss” (101). For example, Lu et al. demonstrated that activation of trogocytosis in intratumoral CTL through the ATF3-CH25H axis dampened the anti-tumor immune response (100). Notably, CD8^+^ T cells engage in cell-to-cell material exchange by obtaining pMHC from APCs or tumor cells in a TCR-dependent manner, may themselves become targets for killing by neighboring CD8^+^ T cells (101, 102). While trogocytosis may prolong antigen receptor engagement and transiently enhance activation, sustained or excessive trogocytosis promote exhaustion (130). From a translational perspective, engineering CAR-T cells to resistant trogocytosis or to avoid the acquisition of inhibitory signals could improve their persistence and therapeutic efficacy in tumors (100, 131).

### 3.4 Cytokines

Multiple studies have elucidated the pivotal roles of cytokine signaling and the inhibitory receptor upregulation in driving CD8^+^ T cell dysfunction within the TME. Cytokines impair CD8^+^ T cell proliferation, cytotoxicity (e.g., granzyme B and perforin expression), and effector functions by inducing exhaustion, metabolic inhibition, and apoptosis. For example, TGF-β and IL-2 suppress CD8^+^ T cell proliferation and cytotoxic activity (103, 104). Mechanistically, TGF-β reduces CXCR3 expression by binding to the CXCR3 promoter through Smad2, thereby diminishing CD8^+^ T cell responsiveness to CXCL10. Ablation of the TGF-β receptor I (ALK5) restores CXCR3 expression, enhances T cell infiltration and cytotoxicity, and promotes tumor regression, these effects are partially reversed by CXCR3 blockade. Furthermore, chronic TGF-β1 signaling orchestrates terminal dysfunction of CD8^+^ T cells through stable epigenetic reprogramming (17). Rebalancing TGF-β1-BMP signaling, for instance with BMP4 agonist SB4, preserves effector-memory programs, reduces exhaustion marker expression, enhances anti-tumor responses, and synergizes with ICB by restoring T cells responsive state.

IL-2 plays pivotal roles in regulating CD8^+^ T cell proliferation, effector function, exhaustion, memory formation, and metabolic adaptability (132). Recent findings underscore the context-dependent effects of IL-2: while elevated IL-2 transiently enhance the proliferation and effector functions of CD25^hi^ CD8^+^ T cells, they also accelerate exhaustion (133–135). In chronic stimulatory settings such as tumor microenvironments, sustained IL-2 signaling drives CD8^+^ T cell exhaustion through STAT5-mediated tryptophan hydroxylase 1 upregulation, generating 5-hydroxytryptophan that promotes inhibitory receptor expression and suppress effector function, revealing a conserved metabolic-epigenetic axis of T cell dysfunction in both mouse and human systems (104). Clinically, high-dose interleukin-2 (HD IL-2) has been employed for the treatment of advanced melanoma and renal cell carcinoma (136, 137), whereas low-dose recombinant human IL-2 selectively modulates the abundance of regulatory T (T_reg_) cells, follicular helper T (T_FH_) cells and IL-17-producing helper T (TH_17_) cells (138). Through these effects, IL-2 promotes the development and survival of T_reg_ cells while inhibiting the differentiation of T_FH_ and TH_17_ subsets, thereby reshaping the immune milieu. Currently, multiple IL-2-based products are under clinical and pre-clinical investigation, requiring evaluation of their effects to reprogram dysfunctional state of anti-tumor CD8^+^ T cells. Modulation of CD8^+^ T cell exhaustion programs by IL-2 to promote the generation of effector cells with stem-like properties provides the immunological rationale for the combination therapy of IL-2 with PD-1 blockade (136, 139). Furthermore, engineered IL-2 partial agonists have been shown to preserve the stem-like properties and mitochondrial fitness of CD8^+^ T cells, thereby enhancing anti-tumor immunity (140). In parallel, IL-6-STAT3 signaling, activated by STK31, also promotes CD8^+^ T cell exhaustion in tumors (105), while IL-18 released in the TME through inflammasome activation drives T-cell exhaustion via IL2-STAT5 and AKT-mTOR signaling downstream of IL-18R (106).

Cytokine pathways also intersect with inhibitory receptor regulation. IL-27 upregulates PD-1 expression via STAT1 signaling yet paradoxically sustains CD8^+^ T cell activity and synergizes with PD-1- PD-L1 blockade (107). Although IL-10 is classically categorized as immunosuppressive through its ability to induce inhibition, recent work suggests that IL-10 alleviates T cell exhaustion by promoting oxidative phosphorylation (OXPHOS) in PD-1^+^ TIM-3^+^ CD8^+^ T cells. An IL-10-Fc fusion protein acts through IL-10 receptors on T cells to specifically enhance OXPHOS, proliferation and cytotoxicity in this subset, thereby reversing exhaustion and enhancing anti-tumor response (108, 109). Conversely, Treg-derived IL-10 and IL-35 cooperatively upregulate the expression of multiple inhibitory receptors and drive BLIMP1-dependent exhaustion of tumor infiltration CD8^+^ T cells, further impeding antitumor immunity (110).

Collectively, these findings underscore the central role of cytokine-mediated signaling networks and inhibitory receptor upregulation in orchestrating CD8^+^ T cell dysfunction within TME, emphasizing the therapeutic potential of targeting these pathways to reinvigorate anti-tumor immunity.

### 3.5 Ions and metabolites (Mg2^+^, Lithium and Ammonia)

The immune function of CD8^+^ T cells is profoundly affected by various ions and metabolites that modulate signaling and metabolic fitness. Magnesium (Mg²^+^) functions as a critical second messenger that regulates CD8^+^ T cell activity through metabolic circuits that sustain effector functions. Deficiency of intracellular free Mg²^+^ impairs NKG2D receptor expression on both NK cells and CD8^+^ T cells, thereby compromising cytotoxic responses against pathogens such as Epstein-Barr virus (111). Lithium, widely used in psychiatric treatment, also exerts immunomodulatory effects on CD8^+^ T cells. Mechanistically, cytoplasmic lactate promotes lysosomal proton influx, meanwhile lithium prevents lysosomal acidification by inhibiting vacuolar ATPase, thereby restoring diacylglycerol-PKCθ signaling to recruit monocarboxylate transporter 1 to mitochondria. This enabled lactic acid transport into mitochondria for CD8^+^ T-cell energy production (112). Ammonia functions as a potent immunosuppressive metabolite within the TME. Elevated ammonia levels reprogram T cell metabolism, leading to exhaustion and proliferation arrest (113). Mechanistically, ammonia accumulation increases lysosomal pH, impairs lysosomal ammonia trapping capacity. This causes ammonia reflux into mitochondria, triggering mitochondrial damage and subsequent cell death (141). Collectively, these findings highlight distinct roles for ions and metabolites in shaping CD8^+^ T anti-tumor immunity.

Indirect suppression in the TME operates through tightly interconnected metabolic, vesicular, structural, and cytokine mediated pathways. These circuits converge CD8^+^ T cells to impair metabolism, signaling, and effector function, driving exhaustion and immune escape. Understanding and therapeutically targeting these mechanisms will be essential for restoring durable anti-tumor immunity.

### 3.6 Integrative strategies to restore T cell function

The progress of immunotherapy has been driven by advances in immune checkpoint research, leading to the clinical approval of adoptive T cell therapy (142, 143). However, CAR-T cell therapies show limited efficacy in many solid tumors and are often linked to immune-related adverse events (144–146). Studies have shown that impaired mitochondrial quality in TILs reduces cytokine secretion and increases the expression of co-inhibitory receptors, while tertiary lymphoid structures in several cancers characterized by chronic inflammatory signaling (147). Moreover, the TME frequently lacks the pro-inflammatory cues or innate immune activation required for optimal T cell priming and expansion, thereby constraining therapeutic efficacy.

To overcome these barriers, emerging strategies aim to synergize innate immune activation with pro-inflammatory stimuli, extending therapeutic benefit beyond checkpoint inhibition, including nutritional interventions (148), oncolytic viruses (149), cGAS-STING agonists (150, 151), cytokine therapy (152), mitochondrial function modulation (153), and vaccine development (149, 154). Addressing metabolic dysregulation, such as lactic acid accumulation in TME (155–157), is particularly critical for maintaining T-cell stemness, emphasizing the importance of mitochondrial fitness in adoptive transfer approaches. Although IL-2 monotherapy showed early promise in metastatic renal cell carcinoma (RCC) and melanoma, its clinical utility was limited by toxicity and Treg activation, prompting a shift toward combination regimens (158, 159). Similarly, pharmacological activation of K^+^ channels, such as with riluzole, a non-specific activator of the KCa3.1 channel, enhances cisplatin uptake in colorectal cancer patients with cisplatin resistance (160).

Improving the efficacy of ICIs requires addressing secondary inhibitory barriers in the TME, including immune-suppressive metabolite accumulation (113), nutrient competition (68), ion imbalances (e.g., high potassium environment), hypoxia, and acidosis-related metabolic hindrances (161, 162). Overcoming these multifactorial constraints is essential for fully unleashing the cytotoxic potential of T cells. Preclinical studies demonstrate that multi-targeted approaches can enhance antitumor efficacy, such as M7824, a bifunctional fusion protein simultaneously targeting PD-L1 and TGF-β (163). In addition, innovative platforms such as nanotube- and exosome-based drug delivery systems (164) and CRISPR-Cas9-based genetic engineering (165) are expanding therapeutic possibilities in personalized gene therapy.

## 4 Discussion

Effective antitumor immunity critically depends on functional CD8^+^ T cells, whose suppression within the TME constitutes a major immune escape mechanism. This suppression occurs through two major routes: (1) Direct cell-to-cell interactions, including tumor cell-CD8^+^ T cell contact (e.g., PD-L1-PD-1), inhibitory signals from CAFs, and immune cell crosstalk (e.g., DC-macrophage-T cell interactions); and (2) Indirect TME-driven mechanisms, such as metabolic competition (nutrient deprivation), intercellular communication (exosomes, tunneling nanotubes, T cell trogocytosis), and microenvironmental perturbations involving immunosuppressive cytokine networks (TGF-β), ionic imbalances (e.g., Mg²^+^ deficiency), and metabolite accumulation (e.g., ammonia).

Within this suppressive networks, CD8^+^ T cell function is progressively impaired by diverse suppressive cues. Recent studies highlight that tumors directly suppress CD8^+^ T cells via inhibitory ligand-receptor interactions, most prominently through the PD-1-PD-L1 axis and the CTLA-4-B7-1 (CD80)-B7-2 (CD86) pathway (56, 86, 166, 167). Additionally, APCs and CAFs suppress CD8^+^ T cell function by engaging CTLA-4 on activated CD8^+^ T cells, thereby constraining the availability of co-stimulatory signals. ICB therapies targeting PD-1-PD-L1, CTLA-4, and LAG-3 have improved survival in multiple cancers (54, 55). However, complete and durable responses remain limited, largely due to tumor heterogeneity, compensatory pathways, and the multifaceted suppressive networks in the TME (56, 168, 169). These limitations underscore the need for complementary or combinatorial strategies that extend beyond classical checkpoint inhibition. A2AR antagonists counteract adenosine-mediated immunosuppression in the TME, thereby restoring T cell-mediated tumor killing (170, 171). Currently, several A2AR antagonists (e.g., AZD4635, CPI-444, AB928) have advanced into Phase II clinical development for indications including prostate cancer and NSCLC (172). Notably, although these candidates vary in developmental stage and tumor type, they demonstrate synergistic effects when combined with PD-1/PD-L1 inhibitors, exhibiting superior antitumor activity compared to either agent alone (171). These findings highlight the potential of targeting metabolic pathways and nutrient competition presents promising avenue to enhance effector responses (169, 173, 174).

Despite these advances, our understanding of how direct and indirect communication networks suppress CD8^+^ T cells in TME remain incomplete. A key challenge lies in decoding these interactions at sufficient resolution, cutting-edge platforms such as spatial resolved transcriptomics, single-cell CRISPR screening (175), nanotherapeutics (176, 177) are now being leveraged to dissect TME-T cell interaction at cellular and molecular levels. Likewise, clinical strategies like CAR-T cell therapy (178, 179) and bispecific antibodies (180) provided translational opportunities for targeting these networks. In particularly, extracellular vesicle-mediated signaling (e.g., exosomes and tunneling nanotubes) represents an underexplored mechanism of tumor-driven immune evasion and a potential strategy of novel therapeutic targets.

Importantly, the functional state of CD8^+^ T cells is tightly dictated by their local microenvironment niche, which is defined by spatial position and communicative interactions with neighboring cells. Ligand-receptor pairs are emerging as critical determinants of these intercellular communication (181, 182). Advances in single-cell and spatial multi-omics allow the dissection of these networks at both cellular and molecularly levels (183). In the parallel, advanced computational framworks enable the systematic analysis of immune infiltration, inference of cell phenotypes, spatial mapping of cellular interactions, and discovery of novel cell-cell communication events, with tools such as CellTalker, PyMINEr, CCCExplorer, SoptSC, NicheNet, CellPhoneDB, CellChat, and CSOmap (184–186).

Another major clinical challenge is the early prediction of immunotherapy efficacy (187). Platforms such as the gel-liquid interface co-culture model have recapitulated human immunity and tumor microenvironment interactions and identified circulating tumor-reactive T cells as biomarkers of treatment response in lung cancers (188). Integration of such ex vivo systems with omics and computational pipelines may accelerate biomarkers discovery.

Therapeutic strategies is increasingly focused on multi-target synergistic interventions (54). Dual-blockade strategies, such as combined PD-1-PD-L1 and TIGIT blockade (189), and tri-blockade regimes, integrating epigenetic modulators (e.g., HDAC inhibitors) with anti-angiogenic agents and PD-1 antibodies, have shown promise in refractory solid tumors by simultaneously remodeling the TME and restoring T cell function (190). Beyond blockade, and emerging therapeutic approach aims to sustain long-term T-cell function by preventing over-activation. An Fc-attenuated LAG-3-TCR bispecific antibody has been engineered to suppress T cell activity independently of MHC-II, demonstrating therapeutic potential in autoimmune models and offering a new avenue for sustaining T-cell function in cancer immunotherapy (191).

Collectively, the intrinsic cellular composition of the TME, coupled with pervasive immune evasion and multifaceted crosstalk, highlights the need for integrative therapeutic strategies that simultaneously target direct inhibitory interactions, metabolic competition, and intercellular communication.

## Glossary

TME: Tumor microenvironment
CTLs: Cytotoxic T lymphocytes
DCs: Dendritic cells
IL-12: interleukin-12
IFN-γ: Interferon-gamma
PD-1: Programmed cell death protein 1
CTLA-4: Cytotoxic T-lymphocyte associated protein 4
Tmp: Memory precursor T cells
Tpex: Progenitor of exhausted T cells
TLS: Tertiary lymphoid structures
TCF1: T-cell factor 1
ICB: Immune checkpoint blockade
Trm: Tissue-resident memory T cells
TAMs: Tumor‐associated macrophages
TGF-βR: Transforming growth factor β receptor
B7-H3: B7 homolog 3
HLA: Human leukocyte antigen
LAG-3: Lymphocyte-activation gene 3
NKG2A: Natural killer group 2 member A
Tregs: Regulatory T cells
MDSCs: Myeloid-derived suppressor cells
CAFs: Cancer-associated fibroblasts
Ceacam-1: Carcinoembryonic antigen-related cell adhesion molecule 1
ZAP70: Zeta-chain-associated protein kinase 70
LSECtin: Liver and lymph node sinusoidal endothelial cell C-type lectin
LUAD: Lung adenocarcinoma
PVRL2: Poliovirus receptor-related protein 2
ERAP1-2: Endoplasmic reticulum aminopeptidase 1-2
NKG2A: Natural killer cell group 2 member A
TILs: Tumor-infiltrating lymphocytes
APCs: Antigen-presenting cells
VISTA: V-domain immunoglobulin suppressor of T cell activation
LRIG1: Ligand for immunoglobulin-like domains 1
FDCs: Follicular dendritic cells
TNF-α: Tumor necrosis factor-alpha
NK: Natural Killer
CCR7: C-C motif chemokine receptor 7
HVEM: Herpes virus entry mediator
BTLA: B and T lymphocyte attenuator
ICIs: Immune checkpoint inhibitors
VEGF: Vascular endothelial growth factor
TNBC: Triple-negative breast cancer
MMP2: Matrix metalloproteinase-2
GLUT1: Glucose transporters glucose transporter 1
AMPK: AMP-activated protein kinase
LDHA: Lactate dehydrogenase
VLCAD: Very-long-chain acyl-CoA dehydrogenase
LCFAs: Long-chain fatty acids
PDA: Pancreatic ductal adenocarcinoma
ARG1: Arginine catabolism by arginase 1
PPARα: peroxisome proliferators-activated receptors
NSCLC: Non-small cell lung cancer
iNOS: Inducible nitric oxide synthase
STAT3: Signal Transducer and Activator of Transcription 3
Mg²⁺: Magnesium
OXPHOS: Oxidative phosphorylation
scTCR-seq: Single-cell T cell receptor sequencing.
